# Potential and Challenges of Aptamers as Specific Carriers of Therapeutic Oligonucleotides for Precision Medicine in Cancer

**DOI:** 10.3390/cancers11101521

**Published:** 2019-10-10

**Authors:** Silvia Nuzzo, Giuseppina Roscigno, Alessandra Affinito, Francesco Ingenito, Cristina Quintavalle, Gerolama Condorelli

**Affiliations:** 1 IRCCS SDN, 80143 Naples, Italy; nuzzo.silvia@gmail.com; 2 Department of Molecular Medicine and Medical Biotechnology, “Federico II” University of Naples, Via Pansini 5, 80131 Naples, Italy; giusy_roscigno@yahoo.it; 3 Percuros BV, Albinusdreef 2, 2333ZA Leiden, The Netherlands; aleaffi4@gmail.com (A.A.); francesco.ingenito@outlook.it (F.I.); 4 Istituto di Endocrinologia ed Oncologia Sperimentale, Consiglio Nazionale delle Ricerche (CNR), 80131 Naples, Italy; 5 IRCCS Neuromed—Istituto Neurologico Mediterraneo, 86077 Pozzilli (IS), Italy

**Keywords:** aptamers, siRNA, miRNA, RNAi, shRNA, ASO, targeted delivery, cancer therapy, diagnosis, brain tumors

## Abstract

Due to the progress made in the area of precision and personalized medicine in the field of cancer therapy, strategies to selectively and specifically identify target molecules causative of the diseases are urgently needed. Efforts are being made by a number of different laboratories, companies, and researchers to develop therapeutic molecules that selectively recognize the tissues and the cells of interest, exhibit few or no off-target and side effects, are non-immunogenic, and have a strong action. Aptamers, artificially selected single-stranded DNA or RNA oligonucleotides, are promising molecules satisfying many of the requirements needed for diagnosis and precision medicine. Aptamers can also couple to their native mechanism of action the delivery of additional molecules (oligonucleotides, siRNAs, miRNAs) to target cells. In this review, we summarize recent progress in the aptamer-mediated strategy for the specific delivery of therapeutic oligonucleotides.

## 1. Introduction

The development of oligonucleotides for therapeutics started two decades ago [1]. Nowadays the treatment of many gene-specific diseases can take advantage of the use of a plethora of oligonucleotides such as microRNAs (miRNAs), miRNA inhibitors (anti-miRs), antisense oligonucleotides (ASOs), and short interfering RNAs (siRNAs). Potentially, these molecules can be designed to target all genes and genome products. Therefore, as therapeutic molecules they have been shown to reduce the expression of genes involved in several diseases, such as cancer. Even if initial studies have induced biotech companies to invest in oligonucleotide-based therapeutics, some limitations to their use, such as their chemistries and the need for specific delivery to target cells, have reduced enthusiasm on their development [2]. For these reasons, investigations aimed at overcoming these limits are needed and should be focused on the study of new delivery systems (viral and non-viral vectors) and on chemical modifications able to increase nuclease resistance, improve pharmacokinetics, and reduce toxicity. One of the major obstacles for oligonucleotide delivery is ascribed to their polyanionic nature, which hinders nucleic acids crossing the lipid bilayer [3]. Various combinatorial strategies have been developed to improve the delivery of oligonucleotides to target cells, such as liposomes [4,5], peptides [6], or nanoparticles [7]. Aptamers are single-stranded DNA or RNA oligonucleotides artificially developed by Systematic Evolution of Ligands by EXponential enrichment (SELEX) [8]. Generally, DNA and RNA aptamers are quite similar in their mechanism of action [9]. DNA aptamers have a better chemical stability and they can be easily generated high-scale in vitro at a cheap price [10]. On the other hand, compared to DNA aptamers, RNA aptamers form more three-dimensional structures, and due to the strong RNA-RNA interaction, they show a high conformation stability [9]. However, RNA aptamers when used in vivo are easily degraded by nuclease digestion. To overcome this issue and to enhance the utility of this class of aptamer for in vivo experimental and therapeutic purposes, some modifications such as 2-fluoro, 2′-amino, and 2′-O-methyl modifications have been introduced in the structure of the RNA aptamer to further increase serum stability [11]. Dassie and colleagues demonstrated in fact that the RNA aptamer A9G is extremely stable to nuclease degradation in 100% human serum over a 1-week period if all the pyrimidines are modified with 2′-fluoro chemistry and all the purines with 2′-O methyl [12].

Due to their tridimensional structure, aptamers bind specific targets with high specificity and affinity. Moreover, they have some crucial advantages, such as low immunogenicity and toxicity, long stability, and low production variability. The high intrinsic target specificity of the aptamers is essential for the development of precision medicine approaches that require a specific targeting of pathological genes. Recent evidences clearly show that aptamers have increased therapeutics effect compared to the conventional therapy, especially in hematological malignancies. For example, in Acute Myeloid Leukemia (AML) the encouraging data on the phase 2 clinical trial of the AS1411 [13,14], a specific aptamer against nucleolin, or in CLL (Chronic Lymphocytic Leukemia) the NOX-A12 [15,16], an aptamer competing to CXCL12 binding to CXCR4, strongly suggest that aptamers can be really translated to the clinic in combination with chemotherapy. Even if there are still a limited number of studies on aptamers, studies on other ODNs (miRNAs, siRNAs and ASOs) have reached more consistent proof of their target specificity and clinical translation.

Nevertheless compared to other ODNs, aptamers not only inhibit their specific target but they are specifically internalized into cells upon binding to their target [17]. This intrinsic property makes aptamers powerful carriers for the specific delivery of secondary molecules such as siRNAs, shRNA, microRNAs, and antisense oligonucleotides (ASOs), overcome the problem of the delivery of iRNAs. 

**Figure 1:**
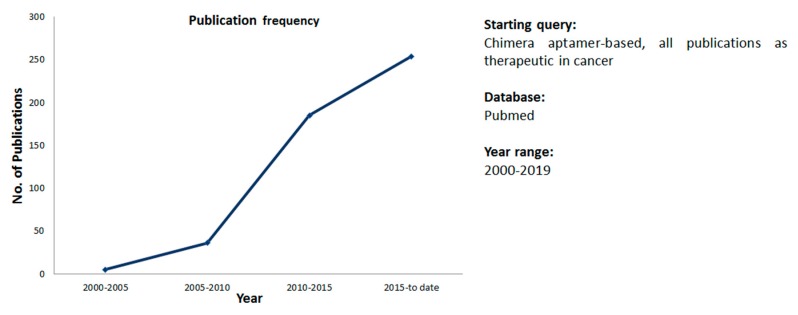
Aptamers chimera in cancer therapy: Publications trend. Pubmed advanced research using the following criteria (((((chimera) OR conjugate) AND cancer) AND aptamer) AND therapy) AND (“from”[Date—Publication]: “to”[Date—Publication]) Sort by: Best Match.

As shown in Figure 1, due to this property of aptamers, there is a rising interest on aptamers as carrier of secondary molecules in the field of cancer treatment. 

**Figure 2:**
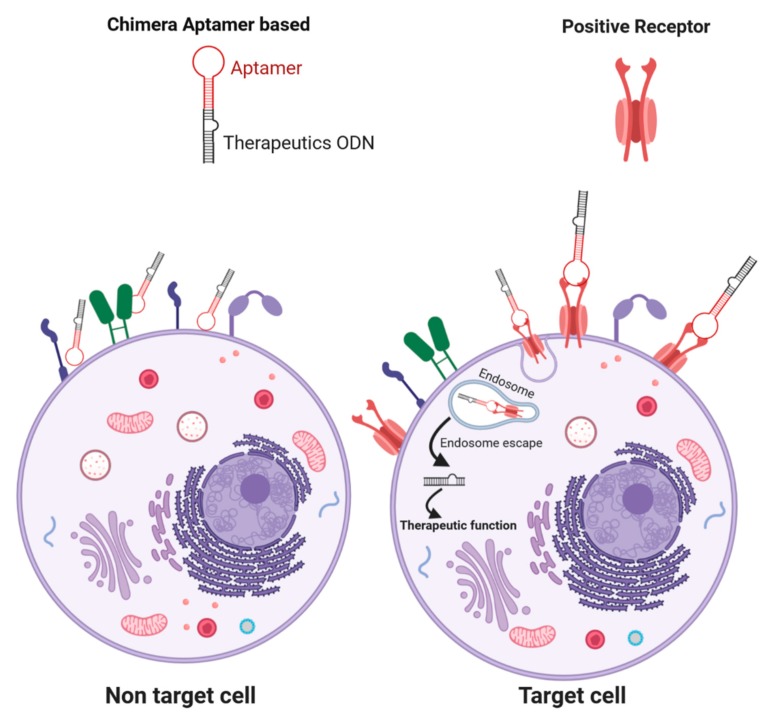
Aptamers deliver ODN. A Schematic design of binding and internalization strategy for ODN delivery.

This review aims to explore the recent advances in the development of a non-viral oligonucleotide delivery system based on aptamers (Figure 2). We have gathered the latest publications supporting them in new delivery strategies in the context of cancer therapy. 

## 2. Aptamers as Carriers for siRNAs 

The RNA interference (RNAi) era, started by Fire and Mello in 1998 [18], completely changed our vision of cell control, basic research, and therapeutic intervention. RNAi is based on short double-stranded RNAs (siRNAs) that downregulate a specific target by degrading mRNA or preventing translation. The RNAi machinery starts from a long double-stranded RNA that is cleaved into a small siRNA (21–23 nucleotide (nt) long) by the endonuclease Dicer. siRNA is then loaded into an RNA-induced silencing complex (RISC), mainly composed by an Argonaute family protein (Ago-2), which cleaves and removes the passenger strand of the siRNA duplex [19]. The single-strand guide RNA, contained in the RISC complex, is then directed to the target mRNA which is recognized by complementary base pairing [20,21]. 

**Figure 3:**
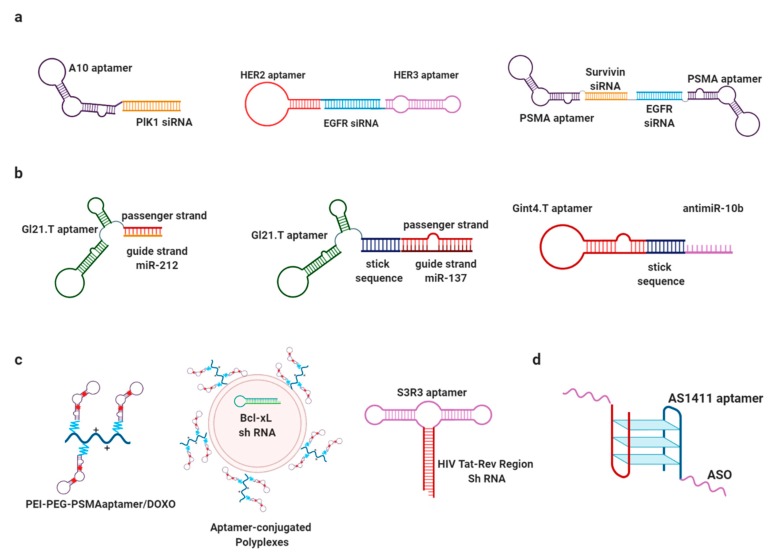
Schematic presentation of principle Aptamer-chimeras. Main examples of the mentioned chimera conjugates (aptamer-siRNA (**a**), aptamer-miRNA/antimiRNA (**b**), aptamer-sh (**c**) and aptamer-ASO (**d**)) are schematically illustrated.

siRNAs represent a clever and efficient method for therapeutic gene silencing, but naked siRNAs are not taken up and internalized into the cells due to their hydrophilic properties, and in biological fluids they are exposed to serum proteins and to endogenous nucleases that do not allow efficient tissue and cell delivery [22]. Moreover, with in vitro delivery strategies, organs such as liver, spleen, and kidney nonspecifically uptake naked siRNAs, altering the biodistribution of the therapeutic siRNA and causing off-target effects [23]. In recent years, to overcome the limits of naked siRNAs, a common approach was conjugation with specific molecules that improve targeted delivery of siRNAs to specific cells or tissues (Figure 3a and Table 1) [24]. In this context, aptamers represent a good strategy for siRNA conjugation in order to increase specific cell delivery and internalization of siRNA molecules. Aptamer-siRNA chimeras (AsiCs), composed by an aptamer and a siRNA or a Dicer substrate siRNA (DsiRNA), bind through the aptamer to a target molecule on the cell surface (e.g., cell receptor, integrin, adhesion molecules), are internalized through endocytosis, and achieve specific delivery. Molecules not degraded by the endosomal compartment are processed by the RNAi machinery, which removes the aptamer portion of the molecule, and induce gene silencing [25]. The first approaches describing an AsiCs were reported in 2006 by Chu TC et al. [26] and McNamara JO et al. [27] using different aptamers against prostate-specific membrane antigen (PSMA). The first group implemented two biotinylated A9 anti-PSMA aptamers and two biotinylated siRNAs against lamin A/C or glyceraldehyde 3-phosphate dehydrogenase (*GAPDH*), while the second group covalently linked to the passenger strand of the siRNAs against polo-like kinase 1 (*plk1*) and *bcl-2* to PSMA aptamer A10′s 3′-terminus and then subsequently annealed the guide strand to the aptamer-siRNA oligo. In both cases, RNAi of the target gene was observed. The authors then more deeply investigated levels of internalization, demonstrating for the first time the possibility of in vivo use of such molecules. The conjugate was further optimized by truncation, adding a two-nucleotide 3′-overhang and a PEG tail, and swapping the siRNA portion. This molecule showed a cytotoxic effect on PSMA-expressing tumors after systemic administration [28]. Furthermore, using the same anti-PSMA A10 aptamer, Wullner and colleagues [29] generated a conjugate able to inhibit Eukaryotic Elongation Factor 2 mRNA (*EEF2*) to specifically induce apoptosis and cell death of prostate cancer cells. Moreover, Liu and colleagues developed the first example of a chimera linking two different siRNAs at the same aptamer portion [30]. With the same PSMA aptamer [28], they created a more complex chimera (named PSEP) composed of a bivalent PSMA aptamer and a dual siRNA against *EGFR* and survivin, two important oncogenes that intersect multiple pathways involved in cancer [31]. This chimera showed the ability to block EGFR-mediated angiogenesis and the metastatic process in prostate cancer. 

Likewise, other molecules have been developed to deliver therapeutic siRNAs to different organs and cells using the specific delivery of the aptamer portion [9]. Indeed, aptamers able to target receptor tyrosine kinases (RTKs), which are specifically expressed in some cancer types, have been used in combination with siRNA to generate AsiCs. In glioblastoma, an AsiC based on the aptamer Gint4.T was able to bind to and antagonize the receptor tyrosine kinase PDGFRβ, and a STAT3-siRNA has been demonstrated able to reduce cell viability and migration in in vitro assays and to inhibit tumor growth in a xenograft mouse model [32]. For breast cancer, a more interesting approach has been used to simultaneously target *EGFR*, *HER2*, and *HER3* using a trivalent chimera made up from an EGFR siRNA positioned between two aptamers able to recognize, respectively, *HER2* and *HER3* (named H2EH3) [33]. This chimera, shown to be poorly immunogenic, easy to produce, highly thermostable, and with a strong biological activity after systemic or intratumoral administration, may represent a new option for treatment of HER2+ breast cancer. In lung cancer, to specifically deliver an anti-nucleolin (NCL) aptamer to lung cancer cells, a chimera composed of two NCL-AsiCs linked together by a hetero-bifunctional crosslinker (sulfo-SMPB) was generated to specifically block snail family zinc finger 2- (SLUG) and neurophilin 1- (NRP1) driven metastatic pathways and epithelial-mesenchymal transition [34].

Recent studies have also shown the feasibility of aptamers carrying nanoparticles encapsulating siRNAs and guiding them to target cells. Zhang and colleagues in 2017 developed a ternary nanocomplex based on an ATP-responsive aptamer duplex to deliver doxorubicin and a Bcl2 siRNA in prostate cancer cells [35]. In another approach, an internalizing B-cell activating factor receptor (BAFF-R) aptamer was conjugated with a sticky bridge to a nanoparticle carrying a Dicer substrate siRNA for STAT3 for the specific targeting of B-cell lines [36]. 

## 3. Aptamers as Carriers of microRNAs

microRNAs (miRNA) were discovered in 1980 [37], and up to now more than 2000 have been discovered. They are located throughout the genome [38] and regulate the expression of one third of the genes in humans, playing a crucial role in many diseases. The biogenesis of miRNAs has been fully characterized. miRNAs are transcribed by RNA polymerase II (pri-miRNA), are cut firstly by Drosha (pre-miRNA) and then by Dicer (mature miRNA, ~22 nt) [39]. The mature guide miRNA is finally loaded into a RISC that directs the miRNA to the mRNA target, promoting its repression [39].

The expression of a large number of miRNAs is dysregulated in cancer [40,41,42], and this is involved in the dysregulation of oncogenes and oncosuppressors. For this reason, miRNA mimics or antisense inhibitors are interesting candidates as therapeutic tools for personalized medicine. One of the most promising candidates is an inhibitor of miR-122 named Regulus (RG-101), currently in phase II trials as an HCV therapeutic [http://www.regulusrx.com/therapeutic-areas/rg-101/]. Other miRNAs have been identified as therapeutic targets for different solid tumors [43]. However, enthusiasm for the potential use of miRNAs as therapeutics has been halted by a difficulty in specific delivery within the cell [44]. For this reason, it is important to find tools able to cross the cellular barrier and improve specificity. Interesting solutions for miRNA delivery have been developed through the use of the aptamers as the carrier (Figure 3b and Table 1) [45]. In the last few years, many aptamer-miRNA conjugates have been designed using covalent or non-covalent approaches and have shown promising results in vitro and in vivo. In 2011, Wu et al. developed therapeutic molecules to treat prostate cancer. By using an RNA aptamer, named A10-3.2, they targeted prostate-specific membrane antigen (PSMA)-positive cells, delivering to these cells the tumor suppressors miR-15a and miR-16-1. Interestingly, the conjugate was also able to promote cancer cell death in vitro [46]. From 2014, the joint groups of de Franciscis and Condorelli described different approaches for the use of GL21.T aptamer as a delivery tool for miRNAs in non-small cell lung cancer (NSCLC) and glioblastoma: the GL21.T aptamer was shown to bind AXL receptor and inhibit its activity [47]. They designed three different conjugates, named GL21.T-let-7g, GL21.T-miR-212, and GL21.T-miR34c, and characterized their function in NSCLC, a tissue in which AXL results overexpressed. Firstly, they characterized the GL21.T-let-7g chimera in vitro and in vivo, demonstrating specific delivery of GL21.T-let-7g to A549 cell lines, upregulation of let-7g miRNA, and, consequently, deregulation of its target HMGA2. The in vitro results were also confirmed in A549 tumor-bearing immunodeficient mice [45]. In a different approach, the GL21.T aptamer was conjugated to miR-34c [48]. The advantage of this new entity was that both moieties (aptamer and miRNA-34c) were able to inhibit AXL receptor, resulting in an enhanced blockade of cell migration and cell viability and increased sensitivity to erlotinib in NSCLC cells [49]. In addition, enhancement of TRAIL sensitivity in AXL-positive NSCLC cells was obtained with the use of the GL21.T-miR-212 chimera thanks to the ability of miR-212 to downregulate PED protein expression, a major player in TRAIL resistance in NSCLC [50,51,52]. More recently, the same groups used a GL21.T-miR-137 chimera to specifically deliver miR-137 to both glioblastoma stem cells (GSCs), reducing GSC number and migration [53] and NSCLC, inhibiting tumor growth in vitro and in vivo [54]. Moreover, Affinito et al., designed two new conjugates based on A40s aptamer able to recognize specifically GBM stem cells and miR-34c and anti-miR-10b, as a new therapeutic approach to treat GBM [55].

To specifically target breast cancer, Rohde et al. developed a chimera, named TRA-miR-126, based on an aptamer for transferrin receptor (TRA), for use as a delivery tool for miR-126, an oncosuppressor miRNA involved in tumor growth, tumor angiogenesis, and metastasis [56]. As part of the chimera, the TRA aptamer delivered miR-126 to positive breast cancer cells, reducing cell proliferation and endothelial recruitment in vitro. Ramezanpour M et al. demonstrated a synergic effect in gastric cancer cells of a chimera consisting of nucleolin-specific aptamer (NCL-Apt) and the miRNA let-7d on JAK2 expression [57].

## 4. Aptamers as Carriers for shRNAs

As mentioned above, aptamers can be successfully adopted for the delivery of miRNAs and siRNAs. Other types of non-coding RNAs, such a short hairpin or small hairpin RNAs (shRNAs), can also be delivered by aptamers and used as therapeutic molecules (Figure 3c and Table 1). shRNAs are RNA molecules with a hairpin-like structure and with a mechanism of action based on RNA interference-mediated post-transcriptional gene silencing of target genes. shRNAs are usually inserted in a vector and transcribed in a duplex structure. After transcription, shRNAs are processed into siRNAs by Dicer [58]. shRNAs can be used to treat several diseases. In cancer therapy, the use of shRNAs can be considered a good strategy because they can produce double-stranded RNA constantly in the nucleus, providing an increase in the efficacy and a decrease in side effects.

As for the other molecules describes above, the main challenge for shRNAs in their complete translation from bench to bedside is delivery; aptamers could help in overcoming this obstacle here too. The first attempt with this strategy was reported by Kim and colleagues, who specifically targeted PSMA-positive prostate cancer cells [59]. Briefly, polyethyleneimine (PEI) was grafted to polyethylene glycol (PEI-PEG) to serve as a platform for Bcl-xL shRNA delivery, and its surface was further conjugated with the anti-PSMA aptamer. Aptamer-conjugated polyplexes (APs) were finally obtained after the intercalation of doxorubicin to form shRNA/PEI-PEG-APT/DOX conjugates. The authors demonstrated that the new AP conjugates selectively delivered both siRNA and doxorubicin to LNCAP cells, resulting in aptamer-mediated binding to PSMA. The AP platform can indeed be a great therapeutic approach for synergistic prostate cancer treatment.

To overcame hormonal responses in prostate cancer, Yang et al. [60] developed a biodegradable poly-dl-lactic-*co*-glycolic acid polymer-based nanoparticle able of encapsulating an androgen receptor (AR) shRNA construct. After shRNA loading, the nanoparticle surface was conjugated with an A10 anti-PSMA aptamer. Interestingly, conjugation with A10 increased the cellular uptake of the nanoparticles in cell lines and mouse models and led to rapid tumor regression.

Moreover, shRNA and aptamer combinations can be used for the treatment of several other diseases. In a recent work by Pang et al. [61], a chimera composed of an aptamer able to recognize HIV integrase was contained in the terminal loop of an anti-HIV Tat-Rev shRNA. This novel approach allowed efficient inhibition of HIV proliferation. This strategy could be potentially extended also to cancer.

## 5. Aptamers as Carriers for ASOs

Antisense oligonucleotides (ASOs) are synthetic single-stranded nucleic acids that alter gene expression by binding RNA [62]. ASOs affect protein levels via several mechanisms. Among them, RNAse H triggering represents the most relevant knockdown system, and via Watson-Crick hybridization, ASOs can form RNA-DNA hybrids that become RNAse H substrates [63,64]. Furthermore, they can modulate pre-mRNA splicing, reducing or restoring protein expression [65]. Since they were discovered in the 1970s [66], ASOs have been deeply investigated and used to specifically alter gene expression in several diseases [67]. Some of them, like Fomivirsen and Nursinersen [68], have been approved by the FDA, and many others are currently under clinical development [69]. Despite the great potential of ASO-mediated therapies, limitations continue to exist because of toxicity, delivery, and uptake issues.

Aptamers have been used to optimize ASO delivery and target engagement, reduce ASO dosage and, accordingly, their off-target effects, and enhance of their safety profile (Figure 3d and Table 1). Kotula et al. recently described a DNA-ASO conjugate with AS1411, a G-quartet DNA aptamer against nucleolin. The aptamer was linked to a splice-switching oligonucleotide (SSO), an ASO subtype able to correct an aberrant stop codon within the Luciferase reporter gene. The AS1411-Luc ASO chimera altered pre-mRNA, restoring Luciferase expression in the target cells. Moreover, this chimera showed a greater effect than the ASO alone, demonstrating the aptamer’s enhancement of ASO efficacy [70]. Similarly, Pei’s group in 2016 produced an aptamer-ASO chimera able to improve the cellular uptake of the ASO by the target cells and to selectively silence galectin-1 expression [71].

The ease with which aptamers can be modified has also been taken advantage of to confer longer half-lives to ASOs. In 2018, Zhu et al. targeted viral NSP9 with short ASOs composed from locked nucleic acids (LNAs), which have a methylene linkage between 2′-O and 4′-C of the sugar ring, This modification led to increased thermostability and resistance to nucleases, allowing improved inhibition of porcine reproductive and respiratory syndrome [72].

## 6. Aptamers: Advantages and Pitfalls as a Delivery System

In general, chemotherapeutic agents to treat cancer have high toxicity both on cancer and healthy cells and usually they are not well tolerated by patients because they have a long list of side effects. Biotechnological advances in the field of drug delivery has led to important medical improvements in the targeting of pathological cells. Specific delivery of drugs allows more effective and precise treatments, with a sharp reduction of dosage and side effects. Thus, the need to have a specific drug delivery system is key to develop a concrete approach for cancer treatment.

Liposomes, viruses, nanoparticles, antibodies and aptamers are all molecules that have been used as delivery vehicles. Liposomes are phospholipidic bilayers organized in vesicular structures and utilized as novel drug delivery system [73]. They have a number of good properties such as biocompatibility, low immunogenicity and toxicity, but also some limitations including loading efficiency and stability and in particular their non-specific adsorption due to their interaction with cell membranes. Nanoparticles have similar limitations, in particular perivascular accumulation and slow release [74]. The main advantage of aptamers relative to these two delivery systems is their target specificity. Indeed, in recent approach aptamers have been used to vehiculate liposomes and nanoparticles in order to overcome their unspecific delivery.

Viruses have been widely used as vectors for gene therapy, but non-viral gene delivery systems have been preferred because viruses have a high risk of carcinogenesis, long-term maintenance, toxin production and immune stimulation [75].

Monoclonal antibodies are the real alternative to aptamers. Antibodies are generated using either animal models or DNA recombinant technologies. The development of recombinant antibodies (rABs) have solved the need of non-animal-derived reagents that show high target recognition specificity [76]. However, antibody size remains ten times higher than aptamer dimension and this significantly reduces vascular permeability and tissues penetration, leading to inadequate antibody distribution and a higher drug resistance [77].

On the other hand, antibodies are well known in therapeutic practice, while aptamers are a new class of molecules and, therefore, they need additional studies aimed to better understand their pharmacokinetic profile and toxicity after systemic administration.

Aptamers show numerous interesting properties such as high specificity, high affinity, and easy synthesis, and for these reasons they are a valid therapeutic tool. In the last years, aptamers targeting pathological cells have been largely developed in the cancer field thanks to their ability to selectively discriminate cancer from healthy cells. Considering that aptamers can have a therapeutic effect, the generation of an aptamer conjugate with enzymes, antibodies, chemotherapeutics, nanoparticles or oligonucleotides could strongly increase their potential. Nevertheless, aptamers, as well as oligonucleotide therapeutics, show limitations that impair their translation to the clinical setting, in particular in vivo stability, pharmacodynamics, renal clearance, and toxicity [11]. Aptamers can be easily modified in order to improve their in vivo stability, overcoming nuclease degradation. Due to their small size, some aptamers are easily removed from the blood stream via excretion by the kidney. Modification such as PEGylation, conjugation with nanoparticles, liposomes or the addition of hydrophilic side chains could increase their molecular weight, reducing renal clearance [78]. However, PEG modification has been shown to induce an allergic response in patients in phase III treated with a REG-1 aptamer (Regado Bioscience) [79,80]. It has also been shown that the use of a multivalent aptamer is able to improve the pharmacokinetics compared to the use of the single aptamer [81]. In this direction, the use of a chimera composed of an aptamer and an oligonucleotide could improve pharmacokinetics and, thus, could allow the clinical translation of oligonucleotides.

A crucial problem, strictly related to aptamer-based chimera molecules, is the amount of chimera that reaches the cytoplasm and acts in the target cell on a specific target. Indeed, only 1% of the chimera-aptamer reaches the cytoplasm. Until now, processing and endosomal escape mechanisms are not totally understood, but to improve the efficacy of the chimera-aptamer compounds it is necessary to enhance the release of the chimera in the cytoplasm. One possible mechanism to overcome this is the conjugation of aptamers to cationic amphipathic peptides or nanomaterials [82]. Moreover, after internalization, any chimera that reaches the cytoplasm must play its therapeutic role based on the ODN used for chimera generation. miRNA, siRNA or shRNAs chimeras are recognized and processed by Dicer into the RISC, which allows the degradation of the mRNA target in a quite conservative mechanism. On the contrary, chimeras composed with ASOs exert their inhibitory activity through different mechanisms. 95% of the inhibitory effect of ASOS occurs via RNAse H-mediated mRNA degradation [63]; other mechanism are (i) the prevention of 5′-capping and 3′-polyadenilation of the pre-mRNA; (ii) the inhibition of protein translation or mRNA splicing, and; (iii) the induction of endogenous micro-RNA degradation [83].

Therefore, to design clinically chimera-aptamer-based therapies, several modifications may have to be introduced to improve the processing of Dicer of miRNAs, siRNAa and shRNAs [28,45] as well as the nuclear penetration of ASOs [84].

## 7. Conclusions

Here, we have summarized progresses in the use of aptamers for non-coding RNA delivery, and we have discussed several examples of recent conjugates (covalent aptamer-siRNA/miRNA/ASO chimeras, non-covalent aptamer-shRNA conjugates and aptamer-nanovector conjugates loaded with shRNAs). However, further challenges must be addressed to facilitate the development of aptamers for cancer therapy, such as the production of shorter sequences with a lower cost, better tumour penetration and greater blood stability. In addition, due to the high cost of large-scale preparation, further developments in aptamer synthesis technologies are needed to allow widespread diffusion of aptamers as therapeutics. Therefore, new innovative aptamer-chimera generation techniques may help to open new scenarios in the future for oligonucleotide-based precision medicine.

## Figures and Tables

**Table 1:** Avaible aptamer-ODN conjugates. Examples of aptamer-chimera conjugates available as therapeutics.

Chimeras Types	Aptamer Chimeras	Disease	References
APTAMER + siRNA	anti-PSMA aptamer-Lamin A/C siRNA	PROSTATE CANCER	[26]
	anti-PSMA aptamer-PLK1 siRNA/ BCL2 siRNA	PROSTATE CANCER	[27]
	anti-PSMA aptamer-EEF2 siRNA	PROSTATE CANCER	[29]
	anti-PSMA aptamer-EGFR siRNA/ survivin siRNA	PROSTATECANCER	[30]
	anti-PDGFRβ aptamer-STAT3 siRNA	GLIOBLASTOMA	[32]
	anti-HER2/HER3 aptamers-EGFR siRNA	BREAST CANCER	[33]
	anti-nucleolin aptamer-SLUG siRNA/ NRP1 siRNA	LUNG CANCER	[34]
	anti-ATP aptamer-BCL2 siRNA	PROSTATE TUMOR	[35]
	anti-BAFF R aptamer-STAT3 siRNAs nanoparticles	LYMPHOMA	[36]
APTARMER + microRNA	anti-Axl aptamer-let-7g	NSCLC	[45]
	anti-PSMA aptamer-miR-15a/miR-16	PROSTATE TUMOR	[46]
	anti-Axl aptamer-miR-34c	NSCLC	[49]
	anti-Axl aptamer-miR-212	NSCLC	[50]
	anti-Axl aptamer-miR-137	GLIOBLASTOMA	[53]
	anti-Axl aptamer- miR-137	NSCLC	[54]
	anti-TRA aptamer-pre-miR-126	BREAST CANCER	[56]
	anti-nucleolin aptamer–let-7d	GASTRIC CANCER	[57]
APTAMER + shRNA	anti-PSMA aptamer- Bcl-xL shRNA	PROSTATE CANCER	[59]
	anti-PSMA aptamer-AR shRNA nanoparticles	PROSTATE CANCER	[60]
	anti-HIV integrase- HIV Tat-Rev shRNA	AIDS	[61]
APTAMER + ASO	anti-nucleolin aptamer-Luciferase SSO	PROSTATE AND PANCREATIC CANCER	[70]
	anti-nucleolin aptamer- Galectin-1 ASO	BREAST AND LUNG CANCER	[71]
